# The impact of lockdown caused by the COVID-19 pandemic on glycemic control in patients with diabetes

**DOI:** 10.1007/s12020-022-02985-1

**Published:** 2022-01-24

**Authors:** Edyta Sutkowska, Dominik M. Marciniak, Karolina Sutkowska, Karolina Biernat, Justyna Mazurek, Natalia Kuciel

**Affiliations:** 1 Faculty of Medicine, Department and Division of Medical Rehabilitation, Wroclaw Medical University, Wroclaw, Poland; 2 Department of Drugs Form Technology, Wroclaw Medical University, Wroclaw, Poland; 3 Faculty of Medicine, Wroclaw Medical University, Wroclaw, Poland

**Keywords:** COVID-19, Diabetes control, Lockdown, Glycated hemoglobin, Glycemia

## Abstract

**Purpose:**

The aim the study was to assess the impact of the lockdown due to COVID-19 on diabetes control.

**Methods:**

The HbA1c value from a pre-lockdown visit (V1) from patients with diabetes was compared to the lockdown visit one (V2) after 3–5 months of its duration. Additional information on how the HbA1c changed and which variables can modify HbA1c during lockdown was also studied.

**Results:**

Records from 65 patients (type 2 diabetes −96,9%) were eligible and revealed that: HbA1c was at the target in 60% of the patients at V2 compared to 40% at V1; HbA1c decreased and normalized in 19, but worsened in 4 participants during the lockdown. No impact on HbA1c of: sex, age, diabetes duration, therapy type and modification before the pandemic, abandonment of the treatment, previous problems with glycemic control, or change in body weight and physical activity during the lockdown, was found. The previous macrovascular complications were the only variable that affected the increase in HbA1c (*p* = 0.0072), OR = 5.33.

**Conclusions:**

The COVID-19 pandemic has not revealed worsened glycemic control in patients with type 2 diabetes, in general. The patients with macrovascular complications turned out to be at risk of the harmful impact of the restrictions on the HbA1c.

## Introduction

Coronavirus disease 2019 (COVID-19) is a disease caused by a new pathogen, severe acute respiratory syndrome coronavirus 2 [1, 2]. The lockdown in Poland caused by the COVID-19 pandemic started on March 24, 2020. This situation has caused a number of urgent changes in normal life, also for chronically ill patients, including individuals with diabetes. In Poland, electronic prescriptions were introduced a few months before, but teleconsultation was not used earlier or it was very rare. Thus, this was the first potential problem for people who were not prepared for such a type of appointments, especially for the elderly, to continue providing them with good care [3]. The second problem which emerged was a restriction which affected their physical activity and activity in the broad sense. For a few weeks, gym-centers, leisure-centers, swimming-pools, etc. were closed but, what was the worst was that for a few weeks also outside walking was forbidden except for shopping or work purposes. It was also recommended that the elderly should not leave their homes and that someone, for example family members, should help them with such a simple activity as shopping. In this way, they were deprived of even the basic (sometimes the only) form of “physical activity” that they previously were involved in. Physical activity, however, is known to have a positive impact on metabolism [4] and it was proven that less active and more obese individuals, such as patients with diabetes type 2, can experience harmful changes which are responsible, for example, for insulin sensitivity and aerobic capacity [5].

The above-mentioned restrictions due to the COVID-19 pandemic also created the next potential problem—people may not have had sufficient influence on the quality of the food that others were buying for them. The pandemic could, therefore, affect their behaviors both by limited burning of calories, including glucose and by providing the body with inferior nutrients (e.g., with a higher content of simple carbohydrates or bad fatty acids).

Additionally, the drop in mood accompanying thef lockdown could make it difficult to fight against bad habits [6]. All of this could have influenced the outcomes of patients with type 2 diabetes, especially as it is characteristic for this group that outcomes are mostly behavior-driven. Thus the patients’ weight and glucose control can change. Normally the effect of such unhealthy behaviors can be detected via e.g., weight control during regular visits by standardized equipment or the glucose level, from a device like e.g., glucometer, can be regularly and directly verified by the medical team. The telemedicine makes it not possible or unreliable.

Of note, obesity, a factor closely connected with improper diet and low physical activity [7], can predispose to a severe course of COVID-19, including a higher risk of death [8]. Unfortunately, individuals with chronic diseases which mostly characterize patients with carbohydrate disturbances, are also much more prone for a more severe course of the disease [9–12].

Since all of the above-mentioned problems can affect glycemia, the aim of our study was to assess the impact of the first months of the lockdown period due to COVID-19 on diabetes control, expressed as HbA1c. To date, no analysis of HbA1c has been performed in individuals with type 2 diabetes during the lockdown. The potential factors contributing to glycemic control were also not examined under the above-mentioned restrictions due to the pandemic.

## Materials and methods

### Place, participants, and main parameter assessment

This is a retrospective study with the approval of the Bioethical Committee of the Wroclaw Medical University (Poland) (approval No. KB-412/2020) which was conducted in NZOZ Nowy Dwor Diabetic Center, Wrocław, Poland.

At the beginning of September, the patients’ records from the Diabetic Center were reviewed for the inclusion criteria: diabetes [13], age ≥18 yo and two available HbA1c values from the defined (see below) period. Pregnancy was an exclusion criterion.

The parameters from a pre-lockdown visit (V1) were compared to ones from a lockdown visit (tele-consultation) (V2). As HbA1c is strictly connected with glycemia during the last 3 weeks to 3 months and is the basis for a definition of poor or good glycemic control, records were analyzed in the context of glycemic control expressed by the HbA1c value and the change in HbA1c between V1 and V2 (see below). This period covered 3–5 months of treatment during the pandemic.

### Definition of the covered lockdown period

The main assumption of our study was that minimum 3 months (April, May and June 2020) of lockdown should have passed, but no more than 5 months (April, May, June, July, and August 2020), whereas the period defined as pre-lockdown covered 1–4 months (December 2019 and January, February, and March 2020) before the restriction was introduced. Thus the maximum gap between the assessment of the pre-lockdown HbA1c and the lockdown HbA1c was no more than 9 months (e.g., patients for whom HbA1c was assessed in December 2019 and August 2020, respectively). The minimum gap was 3 months (patient for whom the first HbA1c was obtained in March 2020 and the second, lockdown one was obtained in July 2020). Data from every adult patient with diabetes who was consulted by the main researcher within the above-mentioned period and met the inclusion criteria mentioned above were analyzed, regardless of diabetes type.

Within the text, for simplicity, the parameters describing the impact of lockdown are also referred to as parameters from 2020 (pre-lockdown or V1 and lockdown or V2) in contrast to a similar period from 2019 (see further part of the text).

### Variables

Other baseline parameters and information, which in the opinion of the researcher could influence the results, were also analyzed as follows:

- patient age and sex,
- level of glycemic control during V1 and V2,
- diabetes duration and type,
- previous problems with diabetes/glycemic control,
- weight change,
- therapy modification during V1
- number of oral agents,
- insulin therapy before V1 and information about its initiation during this appointment,
- acute disease during the lockdown period,
- hospitalization during the lockdown period,
- abandonment of the therapy prescribed during V1,
- maintaining physical activity during the lockdown,
- previous chronic macrovascular complications

### Definitions and criteria for the above-mentioned assessed variables

Glycemic control between V1 and V2 was expressed as the change in the absolute value of HbA1c. The level of glycemic control was defined as good (sufficient) or poor (not-sufficient) with regard to the value of HbA1c available at the appointments. To define “good glycemic control” for each patient, local guidelines were used [13]. The general norm is HbA1c ≤ 53 mmol/mol [7%], but ≤64 mmol/mol [8%] if macrovascular complication/s is/are present. This assessment was additionally incorporated into the analysis to underline clinically important changes, as even if a change of the absolute value of HbA1c is observed, the parameter could still stay within the normal range. Thus not only information about the value of HbA1c was recorded but also the information whether the patient changed his/her status from good-glycemia control to poor-glycemia control or reversely: from poor- to good-glycemia control.

Diabetes duration was based on interviews with patients or the date of the disease onset recorded by a diabetologist if the patients were treated at the aforementioned Center from the beginning. All these information are normally noted in patients’ records.

A previous problem with glycemic control was defined as HbA1c over the individual norm if at least one abnormal result of this parameter was found in a similar period in 2019 (HbA1c at pV1 or pV2). These data could be not available for each patient as an individual could have fallen ill with the disease just before the pandemic or because the parameter was not available for other reasons (another center, no regular appointments).

The evaluation of weight change was based on patient declaration (home-based measurement or patient opinion at V2) and was described as weight gain or steady weight/weight loss in the patient’s opinion. The in-clinical checking was not available during the pandemic time appointment. Also the absolute value of the weight was not recorded for the same reason (no standardization of measurements when using different scales at home, if available at all).

Therapy modification during V1 was defined as a recorded doctor’s decision about additive therapy (higher dose or extra hypoglycemic agent, also initiation of insulin) or reducing the therapy (lower dose or lowering the number of agents).

The number of oral agents was defined as one, two, three or more, including Glucagon-like peptide-1 (GLP-1) receptor agonizts. Insulin intake was also recorded.

Acute disease was defined as the situation when the patient needed help from any physician or surgeon (with or without the need of hospitalization), other than for prescription or minor events (e.g., headache, a minor injury, short-term diarrhea) which did not require a change in diabetes treatment.

Only non-planned hospitalization was recorded.

If the declared treatment at V2 was not consistent with the treatment prescribed during V1, the abandonment of the therapy was marked without the analysis of reasons.

The declaration concerning the level of physical activity during lockdown was made by the patient and recorded at V2 as the same/increased activity or decreased activity.

Chronic macrovascular complications were considered as information about previous: myocardial infarction (MI) or diagnosis of coronary artery disease without MI, stroke, amputation due to ischemia or clinically important peripheral arterial disease (claudication with ankle-brachial pressure index lower than 0.9).

The corresponding author confirms that she had full access to all the data in the study and assumes final responsibility for the decision to submit it for publication.

### Statistical analysis

The study analyzed variables on different scales. For ratio variables (as in Table 1), the following basic descriptive statistics were determined: mean, minimum and maximum values, standard deviation and standard error. The consistency of their distributions with normal distribution was assessed with the use of the Shapiro-Wilk test. Variables whose raw values were statistically significant in an analysis of distribution normality were additionally assessed after their log transformation. Levene’s and Brown-Forsythe tests were used for assessing the homogeneity of variance. All of the analyzed variables met the criterion of variance homogeneity.

**Table 1:** The baseline characteristic for the group and change in patients’ dichotomous parameters during the lockdown

Dichotomous variable	Frequency Tables *N* (%)	
	Yes	No
Sex—women	29 (44.62)	36 (55.38)
T2DM	63 (96.92)	2 (3.08)
Macrovascular complications	13 (20.00)	52 (80.00)
Previous problem with glycemia control	34 (52.31)	21 (47.69)
Patients whose HbA1c increased during lockdown (V2 HbA1c vs V1 HbA1c) *N* = 65	20 (30.77)	45 (69.23)
Patients whose V1 HbA1c was within normal range	24 (36.92)	41 (63.08)
Patients whose V2 HbA1c was within normal range	39 (60.00)	26 (40.00)
Therapy modification at V1	28 (43.08)	37 (56.92)
Weight gain during lockdown	19 (29.23)	46 (70.77)
Maintenance of physical activity during lockdown	61 (93.85)	4 (6.15)
Acute disease during lockdown (number of patients)	5 (7.69)	60 (92.31)
Abandonment of ordered treatment during lockdown	13 (20.00)	52 (80.00)
Patients whose HbA1c increased during last year in similar period (pV2 HbA1c vs pV1 HbA1c) *N* = 42	25 (59.52)	17 (40.48)

*T2DM* type 2 diabetes mellitus, *HbA1c* glycated hemoglobin, *V2* lockdown visit 2020, *V1* pre-lockdown visit 2020, *pV2* visit in 2019 (period similar to V2), *pV1* visit in 2019 (period similar to V1)

For variables in nominal scales (as in Table 2), including dichotomous variables, tables of frequency were calculated.

**Table 2:** The baseline characteristic for the group and change in patients’ continuous parameters before and during the lockdown

Continuous variable	*N*	Mean *µ*	min.	max.	SD
Age	65	70.2	53	85	5.48
HbA1c (V1) % mmol/mol	65	7.5	5.6	13.8	1.22
		58	38	127	9.43
HbA1c (V2) % mmol/mol	65	7.2	5.4	13.7	1.23
		55	36	126	9.40
DM duration years	65	13.6	1.0	41.0	9.07
HbA1c (pV1) % mmol/mol	47	7.2	5.6	10.3	0.94
		55	38	89	7.18
HbA1c (pV2) % mmol/mol	50	7.7	5.4	15.0	1.57
		61	36	140	12.44

*SD* standard deviation, *HbA1c* glycated hemoglobin, *V1* first appointment (immediately before the lockdown), *V2* second appointment, during the lockdown, *DM* diabetes mellitus, *pV1* first appointment in 2019, *pV2* second appointment in 2019

Due to the fact that distributions of concentration values for HbA1c in the analyzed periods of time differed from normal distribution, the following nonparametric tests were used for comparing their mean values: sign test and Wilcoxon’s matched pairs test, and the analysis of variance (ANOVA) for repeated measures designs and Friedman’s post-hoc test. All the variables whose mean values were compared met the variance homogeneity criterion, and their distributions after log transformation partly indicated only minor deviations from normal distribution. Therefore in this case it was considered that it would be justified additionally to present the results of parametric multi-factor/multivariate analysis of variance along with a post-hoc LSD test.

Statistical significance of correlation between variables in nominal scales was assessed with the use of Pearson’s and McNemar’s chi-squared tests for dependent samples, and the correlation between dichotomous and continuous variables was assessed with the use of univariate logistic regression. In the analysis of 2 × 2 contingency tables, odds ratios (OR) were additionally determined along with a 95% interval of significance (±95% CI).

For assessing global correlations between all the analyzed variables, irrespective of their scale, principal component analysis (PCA) was used. The constructed PCA model was estimated with the use of the nonlinear iterative PLS (NIPALS) algorithm. The convergence criterion was determined at 0.00001, and the maximum number of iterations at 100. The number of components was determined by calculating the maximum value of Q^2 predictive capacity using the method of V-fold cross-validation, where V_max = 7. The resultant optimal model of PCA was reduced to two principal components (PC 1 and PC 2).

In all the performed statistical analyses, the assumed significance level was *α* = 0.05.

Statistical analysis was conducted with the use of Statistica 13.01 PL software by StatSoft.

## Results

During the V2 period, 463 teleconsultations were recorded: 39 were marked as Q24 (gestational diabetes) and thus excluded from analysis, but 424 as E11 or E10 (type 2 or type 1 DM). From these 424 visits 156 were found as belonged to the patients who also visited the Center during the defined V1 period (pre-lockdown), but not all of them reached inclusion criteria defined as “available HbA1c from V1 and V2”. Finally, during the above-mentioned period, records from 65 patients were eligible for the analysis (29 from women and 36 from men). The baseline characteristic of the studied group (Table 1) showed that individuals with type 2 diabetes predominated (only one patient suffered from type 1 diabetes and one had diabetes caused by previous pancreatitis) with a range of age typical for this population (Table 2). Women constituted almost half of the group, 1/5 of the patients declared previous macrovascular complications and more than 50% had previous problems with glycemic control, as defined above.

There was a difference between V1 HbA1c and V2 HbA1c (Table 2), with a lower absolute value for the second one (HbA1c decreased during the lockdown), assessed with the use of Wilcoxon’s test (*p* = 0.0027). Despite the fact that in more than 30% of the studied individuals the absolute value of HbA1c increased during the lockdown period, at V2 the HbA1c was within the target range in 60% of the patients compared to only 40% at V1 (*p* = 0.0033). The value of HbA1c normalized in 19, and worsened (not only did it increase but it also reached an abnormal level; according to the definition for the level of glycemic control) in 4 participants during the lockdown period (*p* = 0.0035) and, what is important to note is that none of the patients changed their target for this parameter.

For three of the patients the value of HbA1c did not change between V1 and V2 during the lockdown. For the same number of patients this parameter did not change in a similar period of the preceding year (pV1 vs pV2; HbA1c from pV1 and pV2 was obtained only for 42 participants) and they were not the same participants.

**Fig. 1:**
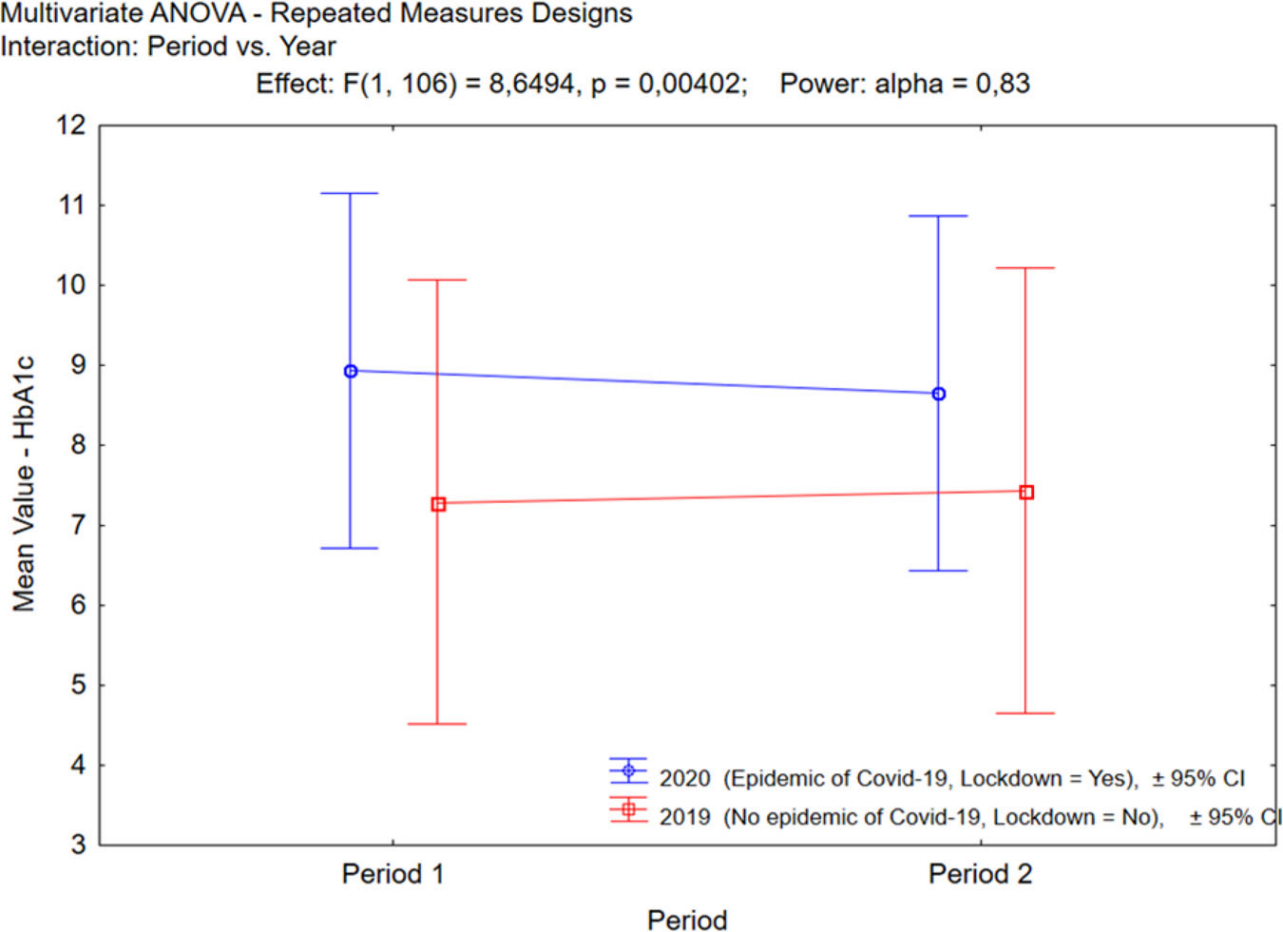
The direction of the HbA1c change when comparing the values for V1 to V2 and pV1 to pV2. *V1-* pre-lockdown visit 2020*, V2-* lockdown visit 2020*, pV1-* visit in 2019 (period similar to *V1*), *pV2-* visit in 2019 (period similar to *V2)*

When comparing two similar periods from 2020 and 2019, we observed an adverse tendency for the change of HbA1c (V1 to V2 and pV1 to pV2) (Fig. 1). The mean HbA1c during the lockdown period (V1 to V2) significantly decreased, and the mean HbA1c for the preceding year (pV1 to pV2) increased (Table 2). The number of patients whose HbA1c value changed during the lockdown was 62 (*p* = 0.0027) from 65, compare to 39 from 42 of the patients whose HbA1c value changed during the similar months of the preceding year (*p* = 0.1410) based on the Wilcoxon’s matched pairs test.

Because of insufficient diabetes control, at V1 more than 40% of the individuals received an extra hypoglycemic treatment. Almost 1/3 of the patients declared weight gain when assessed after a few months of lockdown and almost 100% of the patients declared at least to have maintained the same level of physical activity despite government restrictions. Acute disease was very rare during the assessed period. One-fifth of the respondents declared to have abandoned the previously prescribed pharmacotherapy. The number of, and change in the above-mentioned variables are incorporated in Table 1.

When the entire group of the patients was assessed, we detected no impact of the sex, age, diabetes duration, type of the therapy, declared change in body weight or in physical activity level during the lockdown period on the change in HbA1c. No effect on HbA1c was also found for such variables as therapy modification introduced during V1 or abandonment of the prescribed treatment during the lockdown for the entire group. A previous problem with glycemic control was not an indicator of current (during lockdown) glycemic control, expressed as a worsened HbA1c value during the lockdown period (*p* = 0.08). This means that, for example, individuals with previous poor glycemic control (2019) could reach better control during the lockdown period and vice versa, those with good glycemia control in the preceding year could be found as not-sufficiently controlled patients during the lockdown.

The history of macrovascular complications was the only variable which affected the increase in HbA1c during the lockdown period (*p* = 0.0072) with OR = 5.33.

**Fig. 2:**
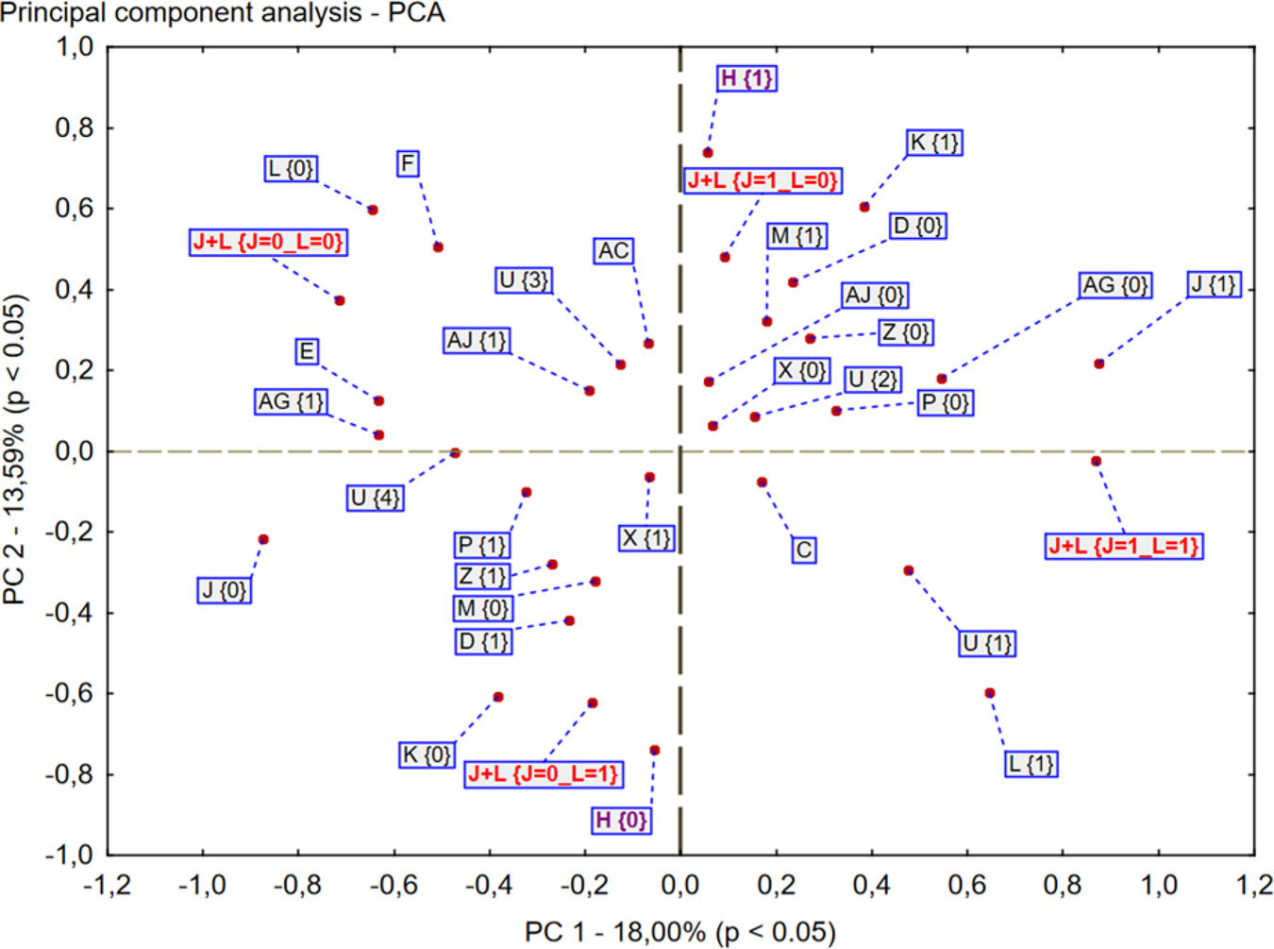
Principal component analysis – PCA. Analyzed variables: *C-*age*, D-*sex (1 = woman), *H*-Patients whose HbA1c(V2) increased vs HbA1c(V1), *J*-Patients whose HbA1c(V1) was within normal range, *K*-Macrovascular complications, *L*-Patients whose HbA1c(V2) was within normal range, *M*-Patients with weight gain during lockdown, *P*-Patients who received additive hypoglycemic treatment at V1, *U*-one oral hypoglycemic agent during 1st visit (just before lockdown), *X*-Acute disease between V1 and V2, *Z*-Abandonment of ordered treatment during lockdown, *AC*-duration (years), *AG*-Previous problem with diabetes control, *AJ*-Patients whose HbA1c(pV2) increased vs HbA1c(pV1); *V1*- pre-lockdown visit 2020, *V2*- lockdown visit 2020, *pV1*- visit in 2019 (period similar to *V1*), *pV2*- visit in 2019 (period similar to *V2*)

We also used PCA to show the potential correlation between the analyzed factors and the two main assessed variables, indicating whether or not the patient had sufficient glycemic control. After taking into account the first variable, marked as J and referred to as “HbA1c within the normal range during V1” (J1 = yes, J0 = no) and the second variable referred to as “HbA1c within the normal range during V2” and marked as L (L1 = yes, L0 = no), the analysis revealed four groups of participants: J1/L0, J0/L1, J1/L1, and J0/L0 which were characterized by different variables (Fig. 2).

The patients from J1/L0, which means the worsening of glucose control during the lockdown period, were mostly treated with two oral hypoglycemic agents (or GLP-1 agonist). For this group of patients, there was a link between the increase of HbA1c and weight gain during the lockdown period, but without statistical significance. We also observed an increase in HbA1c level in the preceding year (2019), within a similar period, mainly for males, in the J1/L0 group.

What was characteristic for the J0/L1 group of participants who improved their glucose control during the lockdown, was that they abandoned the therapy but they also reported a loss of weight. People in this group more frequently took insulin alone or with oral agent/s.

The patients from J1/L1 who had good glycemic control at both V1 and V2 were older, with one, oral (or GLP-1 agonist) hypoglycemic agent and a shorter duration of the disease.

The individuals qualified as the J0/L0 group who had poor glycemic control at both V1 and V2, suffered from diabetes for a longer time, were treated with three or more oral (or GLP-1 agonist) antihyperglycemic agents and had previously problems with good glycemic control (a similar period in 2019).

## Discussion

Published data for the type 1 diabetes population of the patients did not confirm the important impact of the current pandemic on diabetes control [14–17]. Dalmazi et al. [14] underlined that within the pandemic period glycemia can be influenced by age, lower physical activity and stress.

Studies conducted by other authors during the lockdown period have shown that the impact of restrictions may, with varying results, affect individuals with type 2 diabetes. Some of them showed improvement or no change [18–21], and some showed deterioration [22] of diabetes control. In prospective studies, like most of the mentioned above [21–24], when patients are subjected to regular control (sometimes even intervention), an influence of this fact on their behavior, and thus also on the results, is observed. A retrospective assessment in a situation such as a lockdown due to a pandemic, reflects people’s natural behavior and the health impact of sudden changes in a healthcare organization. Also, how diabetes control is expressed in the studies matters. The mere change in blood glucose or HbA1c values may not be sufficient to interpret the potential change in the risk of complications. In the context of metabolic memory, it is important whether the changes in these parameters were so significant that they led to a change in the qualification of diabetes, e.g., from compensated to uncompensated.

The assessment of various variables additionally allows phenotyping patients and dividing them into groups with a greater or lesser potential risk of diabetes decompensation in the future, i.e., during sudden, unexpected changes that we observed in the first months of pandemic. So far, such a complex analysis has not been carried out for this period [25].

With regard to the main parameter—HbA1c, we assessed: the change in its absolute value, the direction of this change (lowering/increasing), and whether the HbA1c of participants is within the normal range. Only such an analysis could give us the true picture of clinically important changes. Our study did not confirm that the first months of the lockdown adversely affected patients’ HbA1c. Moreover, this period seems to be beneficial for this parameter as there were more patients who improved their control than those who worsened it. The absolute value of HbA1c was lower, more patients had the same or lower HbA1c during the lockdown and more of them had HbA1c within the normal range during a control visit after 3–5 months of lockdown (V2), compared to the appointment immediately preceding the lockdown (V1). Because many factors can affect glycemic control, we attempted to find which of them are connected with this observation.

The PCA analysis in our study showed that three variables were linked with better HbA1c, after a few months of the lockdown period: insulin taking, weight loss and abandonment of the therapy by the patient. It is generally known that insulin can improve glycemic control in individuals with type 2 diabetes [26] if they are well educated on how to adjust the dose. It is because insulin gives the patients more flexibility in therapy when behavioral treatment has failed. However, one lockdown study [20] did not confirm this, which contradicts our observation. The loss of weight typically corrects glycemia control and can even reverse diabetes [27, 28]. However, during the lockdown, the lack of exercise as well as unhealthy food choices, promoted weight gain in some observations [18, 29, 30]. Thus it remains an open question why over 70% of our patients declared to have maintained or decreased their body weight. It is possible that individuals who are restricted in one area (e.g., physical activity) become more willing to follow other recommendations (e.g., diet), for fear of worsening their results (e.g., glucose control or weight gain in population with diabetes). The indication of such a tendency comes from the observation dedicated to morbidly obese patients [30] and a systematic review [31]. In the last one the authors pointed that the awareness of the importance of a balanced diet can be an essential factor that may influence dietary choices. However, adherence to dietary recommendations was not analyzed in our retrospective study. It is possible that patients who were restricted in their daily physical activity care much more about their diet or the people who did shopping for them (mostly patients’ relatives) made better choices. Involvement of family members in diabetes management has been confirmed as a positive factor influencing dietary behavior [32, 33].

Of note, the patients who drop out from the previously (at V1) prescribed therapy also lost their weight. This can also potentially explain why “abandonment of the prescribed therapy” was found within variables positively influencing HbA1c value and change—if the control of diabetes is better due to weight lost the dose of medicines can be reduced.

The previous macrovascular complications were the only variable which showed a significant impact on poorer glycemic control in patients whose HbA1c increased. The presence of these chronic diabetes complications increased five times the chance of worsening glycaemia during the lockdown. This observation can be explained by the lockdown alone and has given us the opportunity to define a subgroup of adult individuals with diabetes who need special attention during the COVID-19 pandemic because of a rapid deterioration of glycemia.

The PCA analysis also revealed that a weight gain as well as therapy based on two oral hypoglycemic agents (or GLP-1 agonist) were connected with worse glycemic control. Normally, when glucose rises in patients who use only oral agents, insulin should be started, and this was limited by the lockdown. The previously increased HbA1c level during a similar period of the preceding year in the male group was also associated with the rise of HbA1c during the lockdown. This may rather indicate a factor other than the lockdown, for instance, the impact of the seasons should be considered [34], but it generally remains unclear mainly that was found only for male sex. A long duration of the disease and previous problems with good glycemic control preached a lack of good control both before and during the lockdown what is in line with the other study [22]. To the contrary, a mild course of the disease (characterized by older age and short duration of the disease, with always good glycemic control based on one oral agent) was connected with good glycemic control before and during the pandemic as it represents less ill patients.

In our opinion, the following issues can constitute the study limitations: information obtained from one center only and some of them based on patients’ declarations (body weight, physical activity, acute disease). It also should be considered that the results represent only the initial months of the pandemic and that even during such a short period the restrictions were gradually lifted. However, the population-representative for the country and data collected by one attending physician which excludes the influence of different views on patients’ treatment should be mentioned as the strengths of the study.

## Conclusions

The first months of lockdown due to the COVID-19 pandemic have not revealed worsened glycemic control in patients with type 2 diabetes, but macrovascular complications were found as a variable that put the patients, to be at risk of the harmful impact of the imposed restrictions, even in such a short time. Those results may be useful in identifying patients who deserve closer attention in the case of a lockdown and are mostly interested in help with their glycemic control when behavioral intervention fails.

## Data Availability

Database: https://pl.padlet.com/edytasutkowska/n4us5393iuwb0k8c.
